# Digital Image Decoder for Efficient Hardware Implementation

**DOI:** 10.3390/s22239393

**Published:** 2022-12-01

**Authors:** Goran Savić, Milan Prokin, Vladimir Rajović, Dragana Prokin

**Affiliations:** 1 School of Electrical Engineering, University of Belgrade, 11000 Belgrade, Serbia; 2 The School of Electrical and Computer Engineering of Applied Studies, 11000 Belgrade, Serbia

**Keywords:** digital image decoder, efficient hardware implementation, image decompression

## Abstract

Increasing the resolution of digital images and the frame rate of video sequences leads to an increase in the amount of required logical and memory resources necessary for digital image and video decompression. Therefore, the development of new hardware architectures for digital image decoder with a reduced amount of utilized logical and memory resources become a necessity. In this paper, a digital image decoder for efficient hardware implementation, has been presented. Each block of the proposed digital image decoder has been described. Entropy decoder, decoding probability estimator, dequantizer and inverse subband transformer (parts of the digital image decoder) have been developed in such way which allows efficient hardware implementation with reduced amount of utilized logic and memory resources. It has been shown that proposed hardware realization of inverse subband transformer requires 20% lower memory capacity and uses less logic resources compared with the best state-of-the-art realizations. The proposed digital image decoder has been implemented in a low-cost FPGA device and it has been shown that it requires at least 32% less memory resources in comparison to the other state-of-the-art decoders which can process high-definition frame size. The proposed solution also requires effectively lower memory size than state-of-the-art architectures which process frame size or tile size smaller than high-definition size. The presented digital image decoder has maximum operating frequency comparable with the highest maximum operating frequencies among the state-of-the-art solutions.

## 1. Introduction

The development of new and improvement of existing techniques for the compression and decompression of digital images and videos is very topical today. There is a constant need to improve the quality and resolution of the digital image and the need to increase the frame rate and the duration of the video sequences. All of this results in an increase in the amount of required logical and memory resources for digital image and video processing and storage. Therefore, the improvement of existing techniques and the development of new techniques and hardware architectures for digital image and video compression and decompression, which will decrease the amount of required logical and memory resources, are the only answers to these challenges and many efforts are directed towards achieving that goal. Hardware implementation of 3-D DCT based image decoder with two algorithms to reduce the number of computations and the amount of utilized hardware resources has been presented in [1]. A flexible, line-based JPEG 2000 decoder with customizable level of parallelization without need to use external memory, has been described in [2]. FPGA implementation of a high-performance MPEG-4 simple profile video decoder, capable of parsing multiple bitstreams from different encoder sources has been proposed in [3]. Architecture design of an H.264/AVC decoder described in [4], allows efficient FPGA implementation. A flexible hardware JPEG 2000 decoder for digital cinema, presented in [5], intended for implementation in a single FPGA device, requires a reduced amount of logic and memory resources. A hardware JPEG 2000 decoder architecture based on the DCI specification, which can decode digital cinema frames without accessing any external memory, supports the decoding process in accordance with the order of output images, with reduced storage resources for middle states and temporary image data, has been proposed in [6]. Design and implementation of an efficient memory video decoder with increased effective memory bandwidth has been presented in [7]. FPGA implementation of a full HD real-time high efficiency video coding main profile decoder, solving both real-time and power constraints, has been proposed in [8]. Hardware implementation of a full HD capable H.265/HEVC video decoder, presented in [9], targeted constraints related to hardware costs. Video decoder implemented on FPGAs using 3 × 3 and 2 × 2 networks-on-chip, with communication between the decoder modules performed via a network-on-chip, has been described in [10].

**Figure 1:**
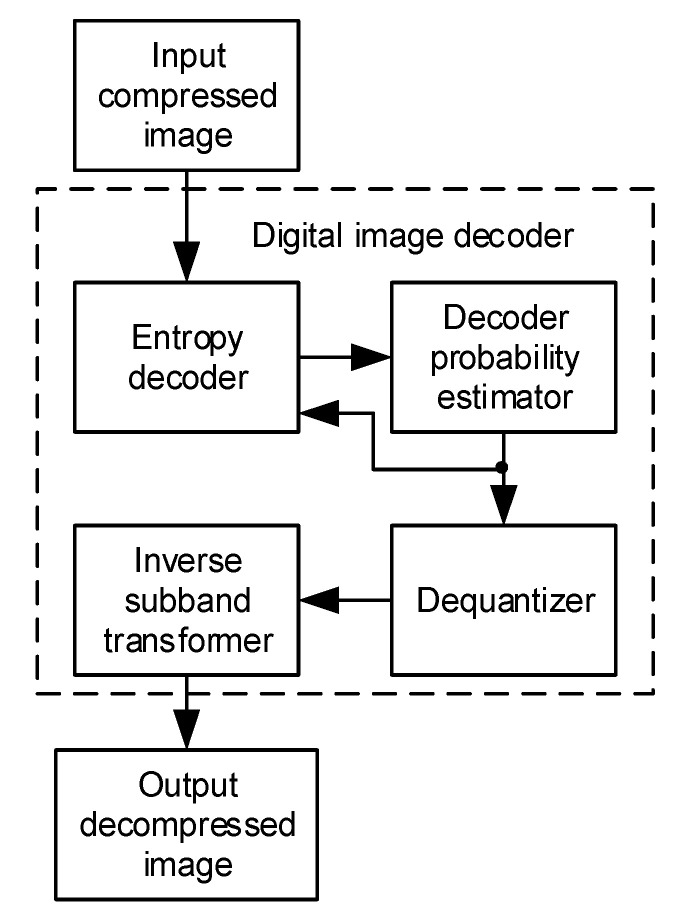
The block diagram of the state-of-the-art digital image decoder.

The block diagram of the state-of-the-art digital image decoder is shown in Figure 1. It consists of entropy decoder, decoding probability estimator, dequantizer and inverse subband transformer. The input compressed image is primarily received and processed by an entropy decoder which forwards its output data to the decoding probability estimator. The decoding probability estimator reconstructs the symbol probabilities within the specified contexts, sends them to the dequantizer and feeds them back to the entropy decoder. These data samples are processed by the dequantizer, which produces dequantized data samples in case of lossy compression or only forwards received data samples to the inverse subband transformer in case of lossless compression. Inverse subband transformer performs inverse filtering and composition of data samples received from the dequantizer and generates pixels of the output decompressed image at its output. As it has been shown in [11,12,13], the arithmetic coding ensures the highest compression ratio, which can theoretically remove all redundant information from the digital message. Arithmetic Q-coder has been presented in [14,15,16,17] and arithmetic Z-coder has been described in [18,19,20,21]. In the well-known JPEG 2000 still image compression standard, the MQ arithmetic coder is used, which is similar to QM-coder adopted in the original JPEG image compression standard described in [22,23,24]. The inverse process of the range encoding presented in [25], has been adopted as the basis of the decoding process implemented in the hardware realization proposed in this paper. The decoding process proposed in this paper is performed for every level of composition and every subband separately. Due to its high performance, uniform scalar quantizer with dead-zone [26] is used for quantization purposes very often. For that reason, it is adopted as a basis for the hardware realization of the dequantizer proposed in this paper.

The inverse subband transformer within the proposed digital image decoder is based on two-dimensional (2-D) discrete wavelet transform (DWT) with Le Gall’s 5/3 filters, which is also a part of the JPEG 2000 still image standard, due to its very good performances. The state-of-the-art hardware architectures of the 2-D DWT are mainly convolution-based or lifting-based. Convolution-based hardware architectures [27,28,29,30,31,32] are usually more complex and utilize larger amount of logic and memory resources. Lifting-based hardware architectures [33,34,35,36,37,38] are usually simpler, have lower computational complexity and utilize less amount of logic and memory resources. The most efficient hardware architectures of the 1-D DWT and 2-D DWT are described in [28] and [36,37,38,39,40,41,42,43,44,45,46,47,48,49,50,51,52,53,54,55,56,57,58,59,60]. The concept of the proposed 2-D DWT with 5/3 filters and its hardware architecture are presented in [61,62,63].

The digital image decoder for efficient hardware implementation presented in this paper has the same block diagram at the highest level of hierarchy as the state-of-the-art decoder shown in Figure 1. However, internal blocks of the proposed digital image decoder have been developed with intention to reduce the amount of utilized memory and logic resources and to optimize the hardware architecture of each internal block and to optimize the hardware architecture of the entire digital image decoder. Some initial research results, related to this topic, have been presented in [64].

This paper has the following structure: Section 2 describes the proposed entropy decoder and decoding probability estimator. The proposed dequantizer is presented in Section 3. Description of the proposed inverse subband transformer, based on two-dimensional (2-D) DWT, can be found in Section 4. Section 5 contains synthesis results of the hardware realization of the entire digital image decoder proposed in this paper. A brief conclusion is presented in Section 6.

## 2. Entropy Decoder and Decoding Probability Estimator

Hardware realization of entropy decoder and decoding probability estimator presented in this paper is based on the inverse process of the range encoding described in [25,65]. Decoding process is performed for every level of composition and every subband separately.

During the process of image compression, the samples of the components of the decomposed signal C (generated by direct subband transformer) had been split into magnitude M and sign S pairs:

(1) M=|C|,

(2) $$S=\left\{ \begin{array}{l} 0,\;C>0\\ 2,\;C=0\\ 1,\;C<0 \end{array}\right..$$

Magnitudes were then classified into magnitude-set indexes MS, which contained a group of magnitudes with similar values. A residual R had been defined as the difference between magnitude M and the lower limit of the sample of the component of the decomposed signal M_lower_limit:

(3) R=M−M_lower_limit.

Magnitude-set indexes MS and the lower limits of the samples of the components of the decomposed signal M_lower_limit are determined based on Table 1.

**Figure 2:**
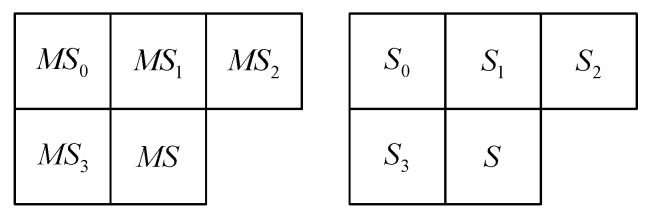
Neighboring magnitude-set indexes $MS_{i}$ and signs $S_{i}$ of already encoded data samples.

In further process of image compression, MS, S and R had been separately encoded. In order to obtain a higher compression ratio, symbols had been defined based on contextual model which contained neighboring data samples, as shown in Figure 2. These contexts are also used in the process of decoding as a part of image decompression.

**Figure 3:**
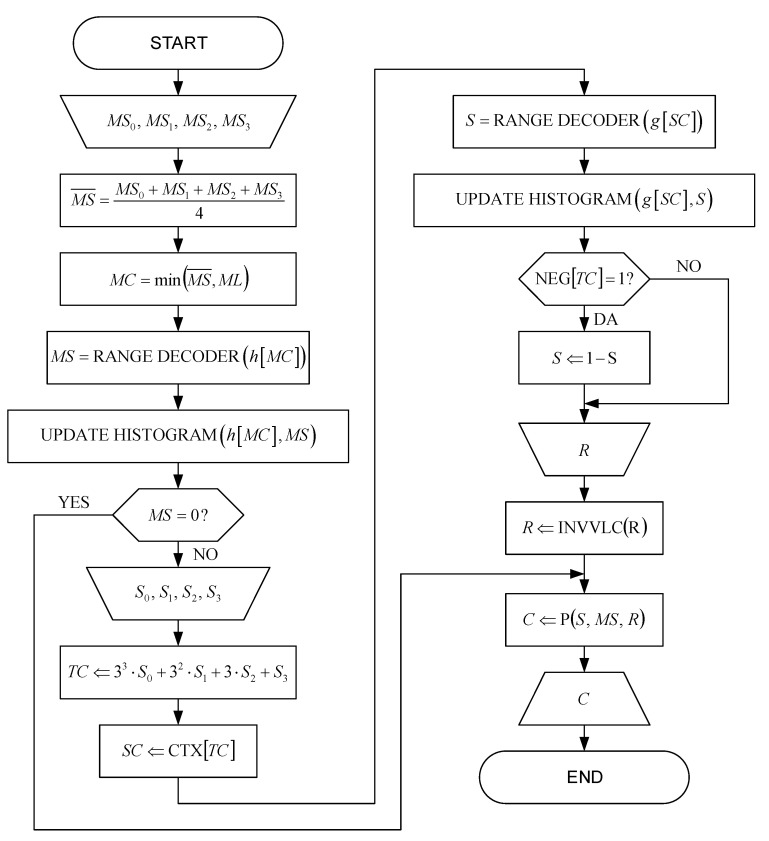
The flowchart of the entropy decoder and the decoding probability estimator.

A flowchart of the entropy decoder and the decoding probability estimator, based on single-pass adaptive histograms with fast adaptation, is shown in Figure 3. The adaptation process starts from a uniform distribution and requires several data samples to complete. The adaptation time is proportional to the number of histogram bins and the difference between the uniform distribution and the exact distribution of the variable being decoded.

First, the values of the neighborhood magnitude-set indexes $MS_{i}$ (shown in Figure 2) of already encoded samples of the components of the decomposed signal are loaded and their mean value $\bar{MS}$ is calculated. Based on the calculated value $\bar{MS}$, the magnitude context MC,which represents the index of the appropriate adaptive magnitude histogram h[MC], is determined, which is then used for the decoding of the magnitude-set index MS using the range decoder. The magnitude context MC is limited by a constant ML, with preferable value ML=4, because the local variance can increase significantly near the sharp edges in the image, which would lead to a large number of histograms and their slow adaptation.

The number of magnitude histograms MH, i.e., the number of different magnitude contexts MC, is preferably limited to MH=ML+1=5. After decoding the magnitude-set index MS, the magnitude histogram h[MC] is updated.

In case of MS=0, sign S is not decoded at all. In case of MS≠0, the neighborhood sign values $S_{i}$ (shown in Figure 2) of already encoded samples of the components of the decomposed signal are loaded and then used for the decoding of a ternary context TC.

Based on the ternary context TC, the sign context SC is then determined using the CTX table represented as Table 2. The CTX table translates 81 different values of ternary contexts TC into a preferable number of five different values of sign context SC for each of the subbands, because a large number of different sign context SC values would lead to histograms that do not adapt at all, which also represents the number of sign histograms SH. This very small number is justified by the fact that the more probable sign S is decoded, which is assured by appropriate examination of the sign and, if necessary, by inversion of the sign S using the NEG table represented as Table 3. Ternary contexts TC with NS=NEG[TC]=0 correspond to a higher probability of a positive sign P(0) than a probability of a negative sign P(1). Ternary contexts TC with NS=NEG[TC]=1 correspond to a higher probability of a negative sign P(1) than the probability of a positive sign P(0).

The sign context SC represents the index of the appropriate adaptive sign histogram g[SC] which is then used for decoding the sign S using a range decoder. After decoding the sign S, the sign histogram g[SC] is updated.

After that, the encoded value of the residual is loaded and decoded using a decoder with a variable length code (INVVLC). Based on the already decoded values of the magnitude-set index MS, using Table 1 given for 16-bit values of the samples of the components of the decomposed signal, the lower limits of the samples of the components of the decomposed signal M_lower_limit are determined, which are then summed with the decoded value of the residual R, forming the decoded value of the magnitude M, as it is shown in Equation (4).

(4) M=R+M_lower_limit.

Finally, at the very end of the decoding process, the decoded value of the samples of the components of the decomposed signal C is formed based on the already decoded magnitude M and sign S values.

**Figure 4:**
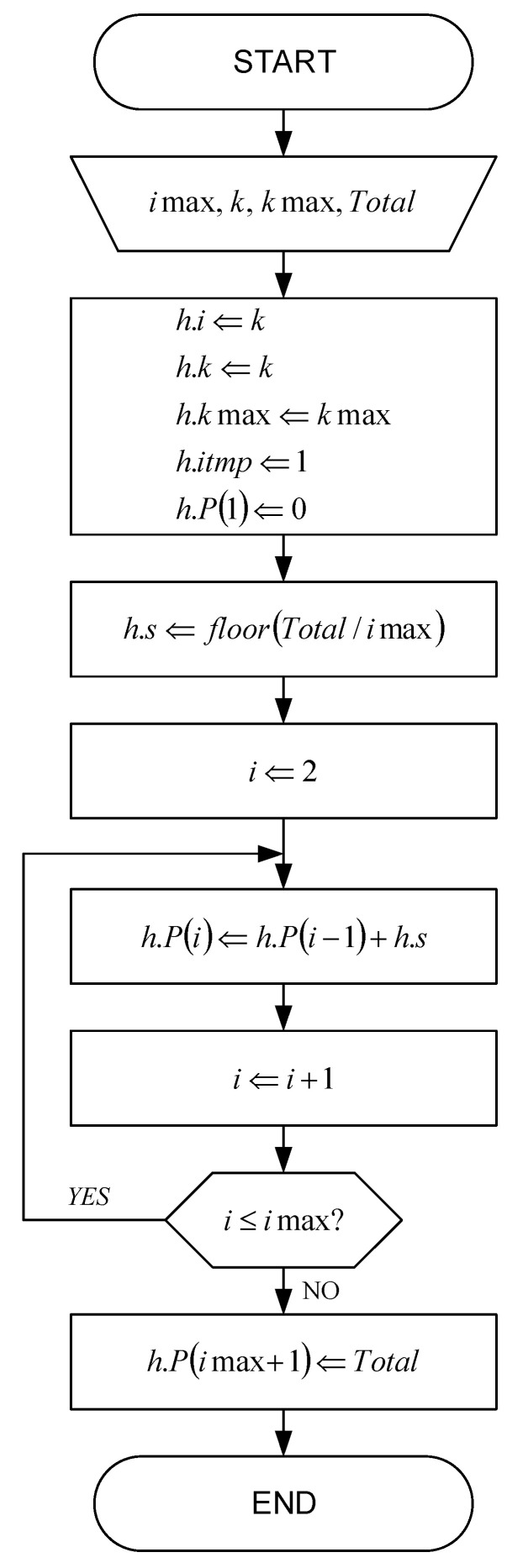
The initialization flowchart for histograms with fast adaptation.

The initialization flowchart for histograms with fast adaptation is shown in Figure 4. Each histogram bin corresponds to a single symbol x, which can be MS for a magnitude histogram or S for a sign histogram. State-of-the-art method for the probability p(x) estimation of an occurrence of symbols x is based on the number u(x) of occurrences of symbol x and the number of occurrences of all symbols Total.

Additionally, it is possible to define the cumulative probability P(x) of all symbols y that precede the symbol x in the alphabet.

(5) $$p\left(x\right)=\frac{u\left(x\right)}{Total},$$

(6) $$Total=\sum_{x}u\left(x\right),$$

(7) $$P\left(x\right)=\sum_{y<x}p\left(y\right)=\frac{U\left(x\right)}{Total},$$

(8) $$U\left(x\right)=\sum_{y<x}u\left(y\right).$$

The main drawback of this simple method is that Total is an arbitrary integer, which means that division operation is necessary in order to calculate the probability p(x). However, in the proposed hardware realization of the entropy decoder and decoder probability estimator, division operation is replaced by shift right operation for w bits, due to:

(9) $$Total=2^{w}.$$

Another drawback of this method is slow adaptation of the probability p(x), due to averaging process. However, in the proposed hardware realization, the adaptation of the probability p(x) is provided by low-pass filtering of the binary sequence I(j) which represents the occurrence of a symbol x in a sequence y of symbols:

(10) $$I(j)=\left\{ \begin{array}{l} 1,\;y\left(j\right)=x\\ 0,\;y\left(j\right)\neq x \end{array}\right..$$

The time response of mentioned low-pass filter is very important, since it is well-known that the bigger time constant of the low-pass filter provides more accurate steady-state estimation, while a smaller time constant provides faster estimation. This problem is especially pronounced at the beginning of the adaptation process, due to a lack of information. In order to avoid making a compromise in a fixed choice of a dominant pole of the low-pass filter, the variation of a dominant pole between minimum and maximum value is implemented.

According to the histogram initialization flowchart shown in Figure 4, the values of the variables are first loaded and, based on them, the variables within the histogram structure h are initialized. In that flowchart, the parameter i represents the histogram bin index, which can have values in the range from 1 to imax. The parameter imax represents the maximum value of the index i of the non-zero histogram, i.e., the total number of different symbols in the alphabet, which is preferably less than or equal to 32 for the magnitude histogram or equal to 2 for the sign histogram. The parameter h.P() represents a string of cumulative probabilities:

(11) $$h.P(i)=P(y|y<i)=\sum_{y<i}p(y).$$

The parameter h.k is a reciprocal of an absolute dominant pole value of the low-pass filter. Variation of its value between h.kmin and h.kmax allows fast adaptation of the histogram after the start. The parameter h.kmax represents the reciprocal value of the minimum absolute dominant pole of the low-pass filter and it is a fixed empirical parameter with preferable value less than Total. The parameter h.kmin represents the reciprocal value of the maximum absolute dominant pole of the low-pass filter and it is a fixed parameter with preferable value h.kmin=2. The total number of symbols within the histogram increased by 1 is represented by the parameter h.i. Finally, the parameter h.itmp represents the temporary value of the parameter h.i before the parameter h.k is changed.

After initializing the variables within the histogram structure h, in accordance with the flowchart shown in Figure 4, the step size h.s is calculated, the index i is initialized and the histogram is initialized. This is followed by incrementing the index i and examining its value. The last step is the initialization of the last histogram bin.

**Figure 5:**
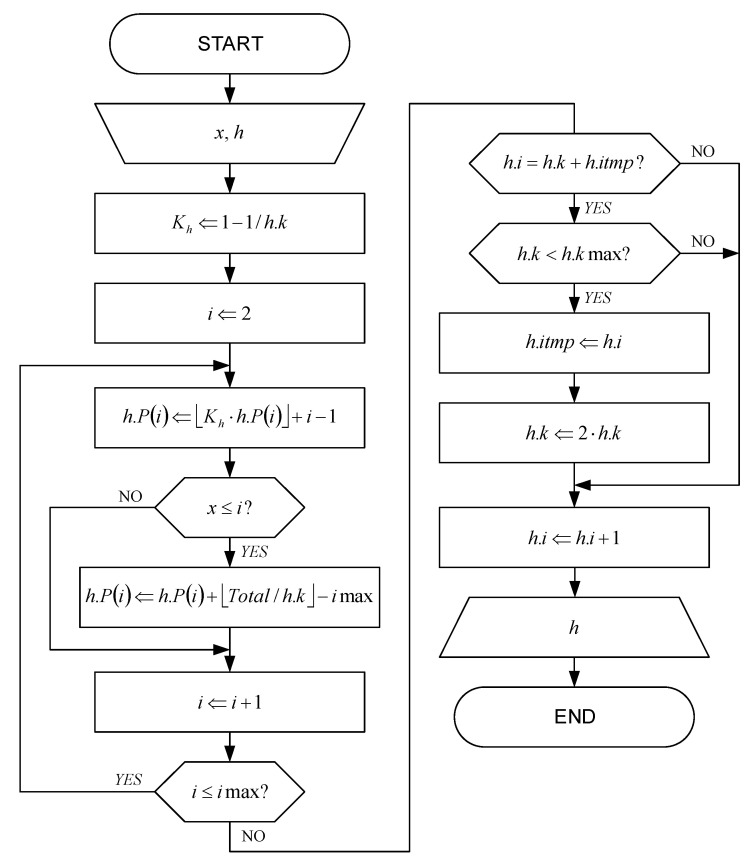
The update flowchart for histogram with fast adaptation.

Figure 5 shows an update flowchart for histogram with fast adaptation, based on the input of the symbol x and already described histogram structure h. Since the range decoder cannot operate with estimated zero probability p(x)=0, even for symbols that do not occur at all, there is a need to modify the binary sequence I(j). Another reason for modifying the binary sequence I(j) is the fact that the modified probability Mp(x)=Total⋅p(x) is estimated using a fixed-point arithmetic. Adaptation of the probability p(x) is performed by low-pass filtering of the modified binary sequence MI(j) defined by Equation (12).

(12) $$MI(j)=\left\{ \begin{array}{l} Total-i\max,\;y\left(j\right)=x\\ \;\;1,\;\;\;\;\;y\left(j\right)\neq x \end{array}\right..$$

The maximum probability maxp(x) and the minimum probability minp(x) can be represented as:

(13) $$\max p(x)=\frac{Total-i\max}{Total}<1;$$

(14) $$\min p(x)=\frac{1}{Total}>0.$$

The preferable low-pass filter is the first order IIR filter in which the divide operation is avoided by keeping the parameter h.k to be the power of two during its variation:

(15) $$Mp\left(x\right)⇐Mp\left(x\right)\cdot \left(1-\frac{1}{h.k}\right)+MI\left(j\right).$$

Instead of updating the modified probability Mp(x), a modified cumulative probability MP(x)=Total⋅P(x) is updated, i.e., a string of cumulative probabilities h.P() is updated. The constant $K_{h}$, which is used for the fast adaptation of histograms, and the histogram bin index i are initialized first. Then, i−1 is added to the cumulative probability h.P(i) prescaled with a constant $K_{h}$, which is equivalent to adding one to a number u(x). This is followed by an update of the cumulative probability h.P(i), only for histograms with the index i greater than or equal to x, which is determined by the previous examination of the values of these parameters.

In the rest of the histogram update algorithm, the histogram is updated according to the following mathematical formulas:

(16) $$h.k=\min\left\lfloor 2^{\left\lfloor \log_{2}\left(h.i+h.k\min-2\right)\right\rfloor },\;h.k\max\right\rfloor ;$$

(17) h.k=max(h.k, h.kmin).

where the preferable value h.kmin=2, which is important for the first h.k during the process of the fast adaptation.

The described method for the fast adaptation of histograms has significant advantages in comparison with state-of-the-art methods. Modifications of estimated probabilities are large at the beginning of the estimation process and much smaller later, which makes possible the detection of small local probability variations, which increases the compression ratio.

**Figure 6:**
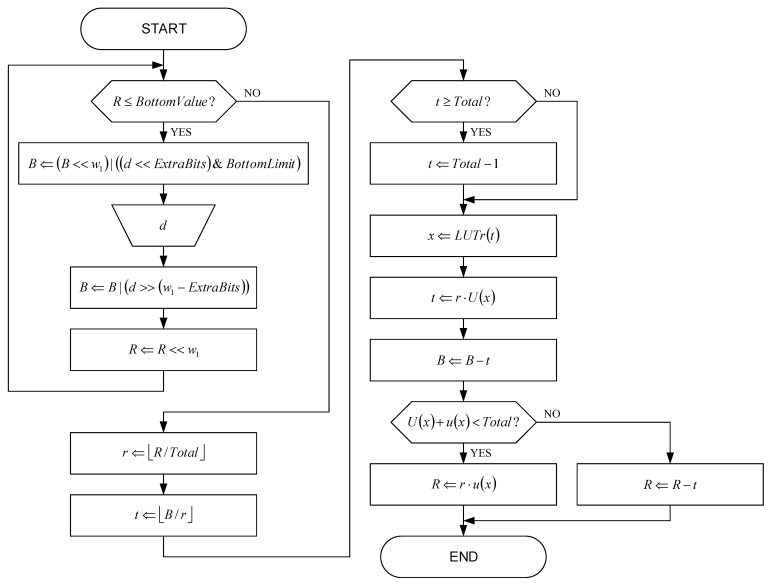
The flowchart of the state-of-the-art range decoder.

Figure 6 shows a flowchart of the state-of-the-art range decoder, which is together with the state-of-the-art range encoder described in [66,67,68]. Decoding is performed using a lookup table LUT (Equation (18)), which is compatible with Equations (19)–(22) for encoding symbol x (the symbol x had been encoded in the buffer of width $s=b^{w}$ in the form of a number i):

(18) $$x=LUT\left(\frac{i+1}{s}\right);$$

(19) i∈(⌊s⋅P(x)⌋, ⌊s⋅(P(x)+p(x))⌋);

(20) ⌊s⋅P(x)⌋≤i<⌊s⋅(P(x)+p(x))⌋;

(21) s⋅P(x)<i+1≤s⋅(P(x)+p(x));

(22) $$P\left(x\right)<\frac{i+1}{s}\leq P\left(x\right)+p\left(x\right).$$

In flowchart from Figure 6, following variables and constants are used:

- B= lower range limit;
- R= range;
- constant $w_{1}$ with preferable value 8;
- constant $w_{2}$ with preferable value 32;
- constant $TopValue=1<<\left(w_{2}-1\right)$ with preferable value 40000000h;
- constant $BottomValue=TopValue>>w_{1}$ with preferable value 00400000h;
- constant $ExtraBits=\left(w_{2}-2\right)\%w_{1}+1$ with preferable value 4;
- constant $BottomLimit=\left(1<<w_{1}\right)-1$ with preferable value 0FFh.

Operators <<, >>, %, | and &, used in that flowchart are borrowed from C/C++ programming language.

Floating point range decoder algorithm after the renormalization and without checking the boundary conditions is described with following equations:

(23) t⇐B/R;

(24) x⇐LUT(t);

(25) t⇐R⋅P(x);

(26) B⇐B−t;

(27) R⇐R⋅p(x).

After introduction of the prescaled range r, the integer range decoder algorithm after the renormalization and without checking the boundary conditions becomes:

(28) $$r⇐\left\lfloor \frac{R}{Total}\right\rfloor ;$$

(29) $$t⇐\left\lfloor \frac{B}{r}\right\rfloor ;$$

(30) x⇐LUTr(t);

(31) t⇐r⋅U(x);

(32) B⇐B−t;

(33) R⇐r⋅u(x);

where:

(34) $$LUTr\left(t\cdot Total\right)=LUTr\left(\frac{B}{r}\right)=LUT\left(t\right).$$

Digits of the symbol x in base b from the input buffer are input. First, the $2w_{1}-ExtraBits$ bits are ignored according to the concept of extra bits. In this particular case, the first byte is a dummy one. Before start of the range decoding process, the following variables need to be initialized:

(35) $$B=d>>\left(w_{1}-ExtraBits\right);$$

(36) R=1<<ExtraBits.

The first part of the range decoding algorithm shown in Figure 6 performs renormalization before decoding, according to the initial examination block. Then, the appropriate bits are written into variable B and new symbol d is input in appropriate input block. After that, the variable B is updated using the appropriate shift operation and the variable R is updated by shifting.

The second part of the range decoding algorithm shown in Figure 6 updates the range. First, the prescaled range r for all symbols is updated using the first division operation. This is followed by deriving the cumulative number of occurrences t of the current symbol using the second division operation, and then limiting the value of t if corresponding condition is met. The next step is to find the appropriate symbol x based on the parameter t value and then to prescale the parameter t value. The parameter B value is updated, followed by the update of the parameter R value using the second multiplication operation with u(x) for the current symbol x for all symbols except the last one. In the case of the last symbol, the parameter R value is updated using the subtraction operation. After the decoding of all data is completed, the final renormalization is performed.

In the state-of-the-art range decoder, the first division operation by Total can be implemented with the shift right operation for $w_{3}$ bits in case when $Total=2^{w_{3}}$, which is provided by the decoder probability estimator. However, the second division operation cannot be eliminated, which contributes to the increasing complexity of the decoder processor because a large number of existing digital signal processors do not support the division operation. Additionally, there are two multiplication operations per each symbol of the compressed image in the range decoder, which contributes to reducing the processing speed in general-purpose microprocessors. These drawbacks have been eliminated in the range decoder described in this paper.

**Figure 7:**
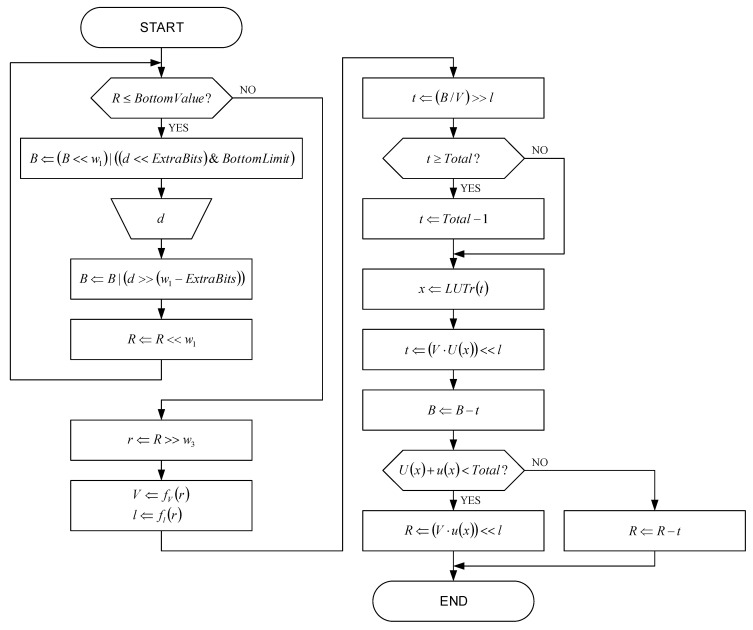
The flowchart of the proposed range decoder.

Figure 7 shows the flowchart of the range decoder proposed in this paper without division operations and, optionally, without multiplication operations. The first division operation by $Total=2^{w_{3}}$ (when calculating the parameter r value) is implemented by the shift right operation for $w_{3}$ bits, due to the fast adaptation of histograms described in this paper. The parameter r is then represented as $r=V\cdot 2^{l}$ and the first multiplication operation is implemented by multiplication with a small number V and shift left operation for l bits in order to calculate the value of the parameter t.

The second multiplication operation is performed when calculating the parameter R value by multiplying with a small number V and shift left operation for l bits. Both small number V multiplication operations are significantly simplified due to the small number of bits used to represent the number V. Furthermore, the multiplication with small, odd numbers, V=3 or V=5, can be implemented by the combination of shift and add operations, which completely eliminates the multiplication operations. The second division operation by r, when calculating the parameter t value, is implemented by the division operation with small number V and shift right operation for l bits. In this case, the division operation by constant small odd numbers V=3, V=5, V=9, V=11, V=13 or V=15 can be implemented with one multiplication operation and one shift right operation according to Table 4, as disclosed in [69,70]. Specially, the division operation by V=7 is the most complex, because it requires the implementation of the addition operation of 049240249h and the addition operation with carry and 0h between the multiplication and shift right operations shown in Table 4.

The approximations used in the implementation of multiplication or division operations in the proposed range decoder led to a smaller compression/decompression ratio. For example, by fixing V=1, it is possible to completely eliminate all multiplication and division operations, but this also causes the largest approximation error and the largest decreasing of the compression/decompression ratio, but not more than 5%. On the other hand, if V is allowed to be V=1 or V=3, the compression/decompression ratio is decreased by less than 1%. Table 5 and Table 6 show the difference in a number of multiplication and division operations per decoded symbol between the state-of-the-art range decoder and the range decoder proposed in this paper. Although approximations, implemented in the proposed range decoder cause a negligible decrease of the compression/decompression ratio and, in contrast, they significantly reduce the hardware complexity of the realization.

## 3. Dequantizer

**Figure 8:**
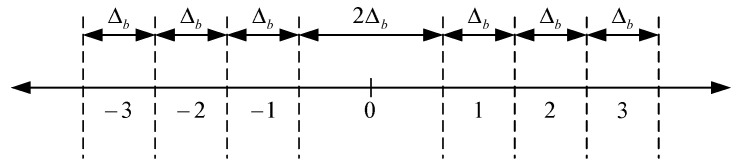
Illustration of the quantization process with uniform scalar quantizer with dead-zone.

Dequantization is only performed in the case of lossy compression, while in the case of lossless compression data samples from the input of the dequantizer are simply routed to its output. Dequantizer proposed in this paper performs the process of dequantization for data samples which had been previously quantized with the uniform scalar quantizer with dead-zone, with quantization step $\Delta_{b}$ and dead-zone width $2\Delta_{b}$, as it is shown in Figure 8.

Generally, each subband b (HH, HL, LH or LL) has its own quantization step $\Delta_{b}$, calculated based on dynamic range of data samples which represent the components of the decomposed signal from subband b. This approach provides higher compression/decompression ratio. Equation (37) describes the quantization process with uniform scalar quantizer with dead-zone:

(37) $$q_{b}=sign\left(y_{b}\right)\;\cdot \left\lfloor \frac{\left|y_{b}\right|}{\Delta_{b}}\right\rfloor$$

where $y_{b}$ represents the component of the decomposed signal from subband b and $q_{b}$ represents the resulted quantized value of data sample.

In order to avoid the division operation and to reduce the hardware complexity of the quantizer and dequantizer, for quantization steps for all four subbands from particular level of decomposition i, the values which represent the power of two are adopted:

(38) $$\Delta_{LHi,HLi}=M\cdot 2^{E-i},\;\Delta_{HHi}=M\cdot 2^{E-i+1},\;\Delta_{LLi}=M\cdot 2^{E-i-1}$$

where M represents the mantissa (integer from the range 64≤M≤127) and E represents the exponent (integer from the range −6≤E≤6).

Dequantized absolute values of data samples which represent the components of the decomposed signal from subbands HH, HL, LH or LL, at level i of composition, are calculated according to the following equations:

(39) $$\left|y_{LHi\_deq}\right|=\left|q_{LHi}\right|\cdot M\cdot 2^{E-i}+M\cdot 2^{E-i-1};$$

(40) $$\left|y_{HLi\_deq}\right|=\left|q_{HLi}\right|\cdot M\cdot 2^{E-i}+M\cdot 2^{E-i-1};$$

(41) $$\left|y_{HHi\_deq}\right|=\left|q_{HHi}\right|\cdot M\cdot 2^{E-i+1}+M\cdot 2^{E-i};$$

(42) $$\left|y_{LLi\_deq}\right|=\left|q_{LLi}\right|\cdot M\cdot 2^{E-i-1}.$$

The hardware complexity of the dequantizer proposed in this paper is significantly reduced, since the multiplication operation by power of two is implemented by using permanently shifted hardware connections between input and output bit lines, and due to multiplication with narrow-range integer M, which is implemented by a simple lookup table.

## 4. Inverse Subband Transformer

Inverse subband transformer is an important part of digital image decoder from the aspect of memory resources utilization. Optimal realization of inverse subband transformer can make important contribution to reducing the capacity of used memory and the neglecting the importance of inverse subband transformer optimization could lead to a significant increase in the amount of utilized memory resources.

The proposed hardware realization of the inverse subband transformer is based on the 2-D DWT with 5/3 filters. Equation (43) describes one-dimensional (1-D) inverse low-pass Le Gall’s 5/3 filter, while Equation (44) describes 1-D inverse high-pass Le Gall’s 5/3 filter:

(43) $$w_{0}[n]=\frac{1}{2}y_{0}[n-1]+y_{0}[n-2]+\frac{1}{2}y_{0}[n-3];$$

(44) $$w_{1}[n]=-\frac{1}{8}y_{1}[n]-\frac{1}{4}y_{1}[n-1]+\frac{3}{4}y_{1}[n-2]-\frac{1}{4}y_{1}[n-3]-\frac{1}{8}y_{1}[n-4].$$

**Figure 9:**
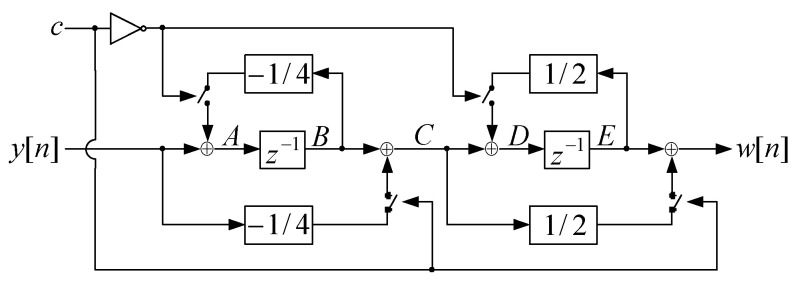
Non-stationary hardware realization of the 1-D inverse 5/3 filter.

The basic building block utilized for 2-D DWT filtering is non-stationary hardware realization of the 1-D inverse 5/3 filter shown in Figure 9.

**Figure 10:**
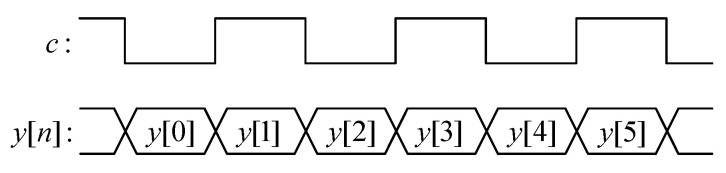
The time diagram of control signal *c* in the proposed 1-D inverse 5/3 filter.

The control signal c controls four switches, providing two different topologies of the filter: one topology for input data samples y[n] with even indexes n=2p and another topology for input data samples y[n] with odd indexes n=2p+1. The control signal c is at low level (c=0) for every input data sample y[n] with even index n when two upper switches are closed, while two lower switches are opened. Control signal c is at high level (c=1) for every input data sample y[n] with odd index n when two upper switches are opened, while two lower switches are closed. The time diagram of control signal *c* in the proposed 1-D inverse 5/3 filter is shown in Figure 10.

**Figure 11:**
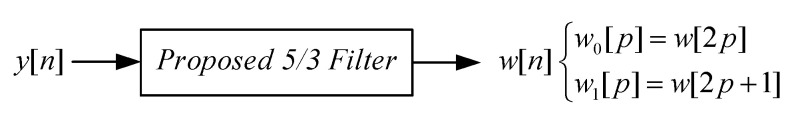
Block diagram of the proposed 1-D inverse 5/3 filter.

The proposed 1-D inverse DWT 5/3 filter provides output data samples for even indexes n=2p and odd indexes n=2p+1 in an interleaved fashion, as shown in Figure 11.

Hardware realization of 1-D inverse 5/3 filter from Figure 9 has been implemented on EP4CE115F29C7 FPGA device from Altera Cyclone IVE family [71]. The synthesis results for the proposed non-stationary filter realization and state-of-the-art convolution-based and lifting-based realizations (implemented on the same FPGA device), obtained using Altera Quartus II 10.0 software, are presented in Table 7.

It can be seen that hardware implementation of the proposed non-stationary 1-D inverse 5/3 filter utilizes the lowest number of total logic elements and registers, has the shortest critical path delay, allows the highest maximum operating frequency and has the lowest total power dissipation in comparison with state-of-the-art realizations.

**Figure 12:**
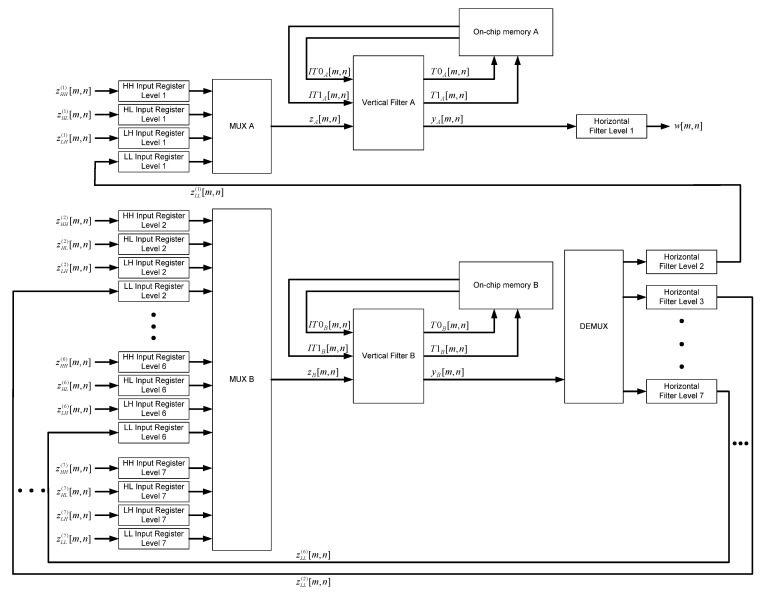
The block diagram of the proposed 2-D inverse DWT 5/3 architecture.

The block diagram of the proposed 2-D inverse DWT 5/3 architecture, with J=7 levels of composition, is shown in Figure 12. The input data samples are the components of the decomposed signal $z_{HH}^{\left(j\right)}[m,n]$, $z_{HL}^{\left(j\right)}[m,n]$ and $z_{LH}^{\left(j\right)}[m,n]$ from level *j* (*j*=1,2,…,7) of composition and the components of the decomposed signal $z_{LL}^{\left(7\right)}[m,n]$ from level 7 of composition. The subband LL represents the data samples produced as the result of forward low-pass filtering over rows and forward low-pass filtering over columns within the direct subband transformer, which is a part of a digital image encoder. The subband HL represents the data samples produced as the result of forward low-pass filtering over rows and forward high-pass filtering over columns. The subband LH represents the data samples produced as the result of forward high-pass filtering over rows and forward low-pass filtering over columns. Finally, the subband HH represents the data samples produced as the result of forward high-pass filtering over rows and forward high-pass filtering over columns.

The input data samples from level 1 of composition are routed through a multiplexer “MUX A” generating data samples $z_{A}[m,n]$ shown in Equation (45), then vertically filtered by “Vertical Filter A”, producing the data samples $y_{A}[m,n]$ shown in Equation (46), which are then horizontally filtered by “Horizontal Filter Level 1” generating the pixels of the reconstructed image w[m,n]. The sequence of data samples $y_{A}[m,n]$ contains high-pass ($y_{H}^{\left(1\right)}[m,k]$) and low-pass ($y_{L}^{\left(1\right)}[m,k]$) data components at level 1 which are to be horizontally filtered.

(45) $$z_{A}[m,n]=\left\{ \begin{array}{l} z_{LH}^{\left(1\right)}[m,k],\;for\;m=2l\;and\;n=2k\\ z_{LL}^{\left(1\right)}[m,k],\;for\;m=2l\;and\;n=2k+1\\ z_{HH}^{\left(1\right)}[m,k],\;for\;m=2l+1\;and\;n=2k\\ z_{HL}^{\left(1\right)}[m,k],\;for\;m=2l+1\;and\;n=2k+1 \end{array}\right.;$$

(46) $$y_{A}[m,n]=\left\{ \begin{array}{l} y_{H}^{\left(1\right)}[m,k],\;for\;n=2k\\ y_{L}^{\left(1\right)}[m,k],\;for\;n=2k+1 \end{array}\right..$$

The input data samples from level *j* (*j* = 2,3,…,7) of composition are routed through a multiplexer “MUX B” generating data samples $z_{B}[m,n]$ shown in Equation (47), and then vertically filtered by “Vertical Filter B”, producing the data samples $y_{B}[m,n]$ shown in Equation (48), which are then horizontally filtered by “Horizontal Filter Level *j*” generating the components of the decomposed signal $z_{LL}^{\left(j-1\right)}[m,n]$ (*j* = 2,3,…,7), which are later used for inverse filtering at level *j* − 1. The sequence of data samples $y_{B}[m,n]$ contains high-pass ($y_{H}^{\left(j\right)}[m,k]$) and low-pass ($y_{L}^{\left(j\right)}[m,k]$) data components at level *j* (*j* = 2,3,…,7) which are to be horizontally filtered.

(47) $$z_{B}[m,n]=\left\{ \begin{array}{l} z_{LH}^{\left(j\right)}[m,k],\;for\;m=2l\;and\;n=2k\\ z_{LL}^{\left(j\right)}[m,k],\;for\;m=2l\;and\;n=2k+1\\ z_{HH}^{\left(j\right)}[m,k],\;for\;m=2l+1\;and\;n=2k\\ z_{HL}^{\left(j\right)}[m,k],\;for\;m=2l+1\;and\;n=2k+1 \end{array}\right.;$$

(48) $$y_{B}[m,n]=\left\{ \begin{array}{l} y_{H}^{\left(j\right)}[m,k],\;for\;n=2k\\ y_{L}^{\left(j\right)}[m,k],\;for\;n=2k+1 \end{array}\right..$$

**Figure 13:**
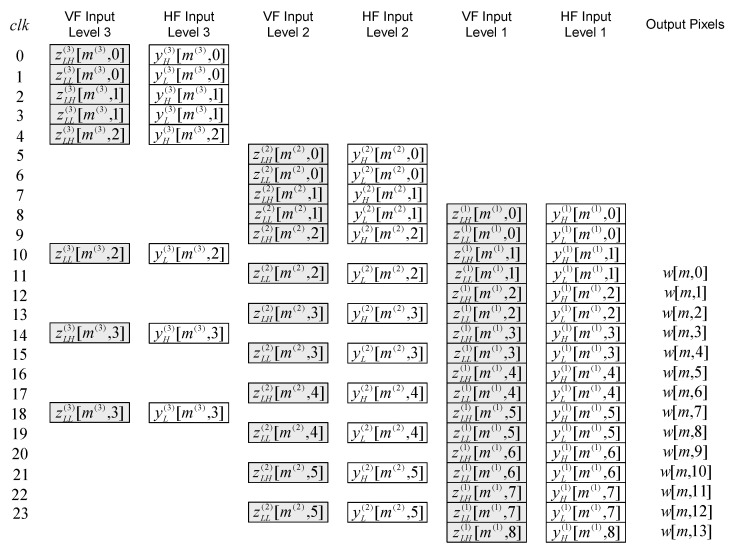
The time diagram of the 2-D inverse DWT 5/3 filtering at the beginning of even lines.

**Figure 14:**
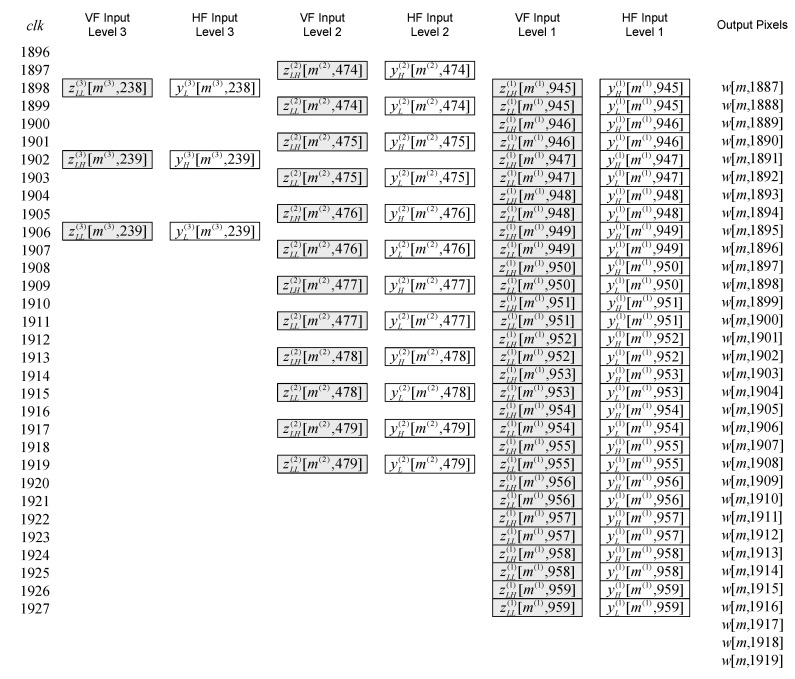
The time diagram of the 2-D inverse DWT 5/3 filtering at the end of even lines.

The time diagram of the 2-D inverse DWT 5/3 filtering at the beginning of even lines (starting from 0) for the first three levels of composition is shown in Figure 13. This pattern continues until the end of the even lines, and time diagram of the 2-D inverse DWT 5/3 filtering at the end of even lines for the first three levels of composition can be seen in Figure 14.

The time diagram of the 2-D inverse DWT 5/3 filtering of the odd lines is almost the same as for the even lines. There are only few differences: every even signal component (starting from 0) at the input of vertical filter belongs to the subband HH ($z_{HH}^{\left(1\right)}[m^{\left(1\right)},n^{\left(1\right)}]$), every odd signal component at the input of vertical filter belongs to the subband HL ($z_{HL}^{\left(1\right)}[m^{\left(1\right)},n^{\left(1\right)}]$) and the first level of composition is also the only level of composition, because the signal components from the subbands HH and HL are not generated based on the signal components from the previous levels of composition.

**Figure 15:**
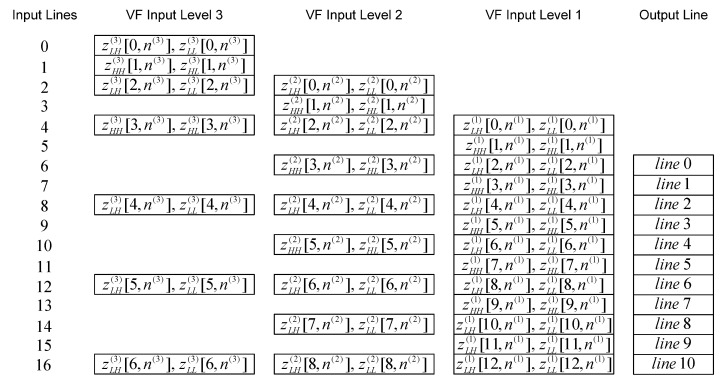
The time diagram of the beginning of the line-wise filtering.

**Figure 16:**
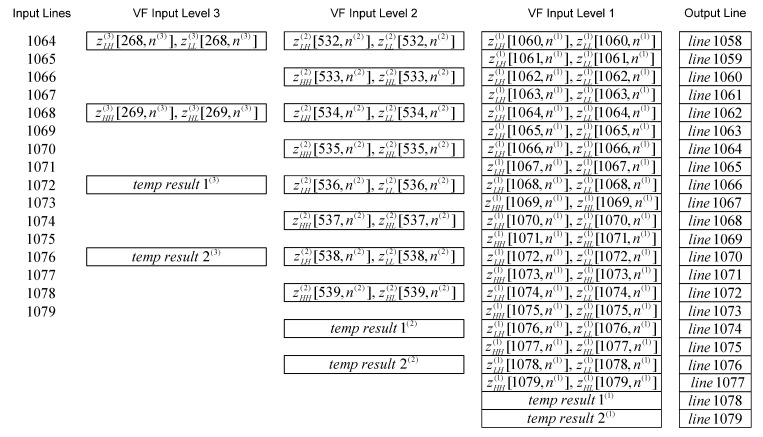
The time diagram of the end of the line-wise filtering.

The time diagram of the beginning and the time diagram of the end of the line-wise inverse filtering for the high-definition resolution image are shown in Figure 15 and Figure 16, respectively. Notation “$z_{LH}^{\left(j\right)}[m^{\left(j\right)},n^{\left(j\right)}]$, $z_{LL}^{\left(j\right)}[m^{\left(j\right)},n^{\left(j\right)}]$”, for even lines (starting from 0) at level *j*, represents the following sequence of signal components: $z_{LH}^{\left(j\right)}[m^{\left(j\right)},0]$, $z_{LL}^{\left(j\right)}[m^{\left(j\right)},0]$, $z_{LH}^{\left(j\right)}[m^{\left(j\right)},1]$, $z_{LL}^{\left(j\right)}[m^{\left(j\right)},1]$, $z_{LH}^{\left(j\right)}[m^{\left(j\right)},2]$, $z_{LL}^{\left(j\right)}[m^{\left(j\right)},2]$, etc. Notation “$z_{HH}^{\left(j\right)}[m^{\left(j\right)},n^{\left(j\right)}]$, $z_{HL}^{\left(j\right)}[m^{\left(j\right)},n^{\left(j\right)}]$”, for odd lines at level *j*, represents the following sequence of signal components: $z_{HH}^{\left(j\right)}[m^{\left(j\right)},0]$, $z_{HL}^{\left(j\right)}[m^{\left(j\right)},0]$, $z_{HH}^{\left(j\right)}[m^{\left(j\right)},1]$, $z_{HL}^{\left(j\right)}[m^{\left(j\right)},1]$, $z_{HH}^{\left(j\right)}[m^{\left(j\right)},2]$, $z_{HL}^{\left(j\right)}[m^{\left(j\right)},2]$, etc. All these signal components are vertically and then horizontally filtered by appropriate inverse filters.

The internal intermediate results ‘temp result 1’ and ‘temp result 2’ at the current level of composition are used for generating the last two lines of resulting signal components from the next level of composition.

**Figure 17:**
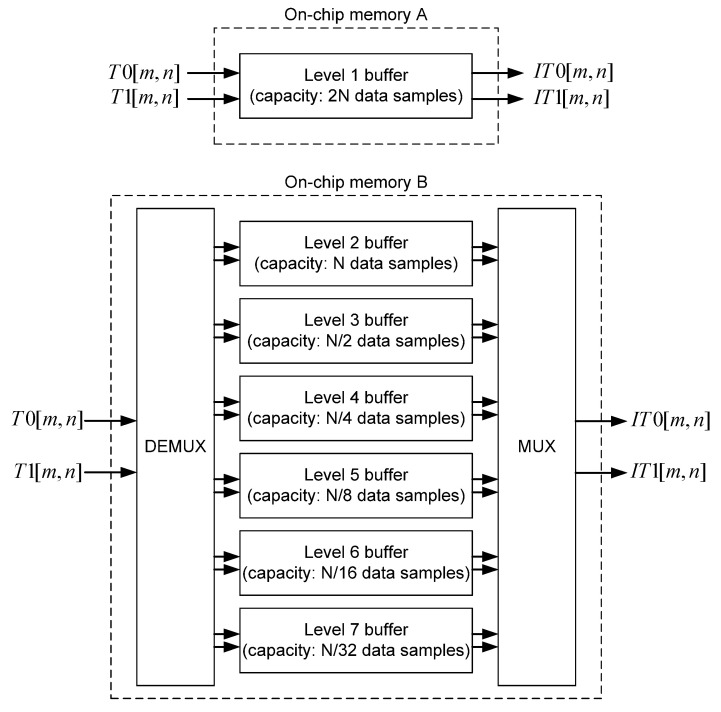
On-chip memory.

In order to ensure the proper inverse 2-D DWT 5/3 filtering of N×N image, two lines of intermediate results have to be stored into on-chip memory (shown in Figure 17) at each level of composition. The intermediate results from level 1 of composition are stored into “On-chip memory A”, which contains one FIFO buffer with capacity of 2N data samples, while the intermediate results from all other levels of composition are stored into “On-chip memory B”, which contains six FIFO buffers (in case of J=7 levels of composition) with capacity halved at every succeeding level, starting from capacity of N data samples at level 2. The total on-chip memory capacity needed for N×N image filtering with J levels of composition is:

(49) $$2N+N+\frac{N}{2}+\ldots +\frac{N}{2^{J-2}}=4N\left(1-2^{-J}\right).$$

Due to the very low capacity of required memory, the proposed inverse 2-D DWT architecture does not require off-chip memory at all. The comparison between the proposed architecture and the best state-of-the-art 2-D inverse 5/3 DWT architectures so far published in the literature, in terms of required capacity of on-chip and off-chip memory, is presented in Table 8. It can be concluded that the proposed 2-D inverse 5/3 DWT architecture, for N×N image and J→∞ levels of composition, requires the total memory capacity of 4N data samples, which is 20% lower capacity compared with the best state-of-the-art architecture.

## 5. Synthesis Results of the Hardware Implementation of the Proposed Digital Image Decoder

Described in this paper is a digital image decoder for efficient hardware implementation with three color planes (Y, U and V) and its functional correctness had been verified by implementation within Altera DE2-115 development board, produced by Terasic Technologies [72], on an EP4CE115F29C7 FPGA device. Synthesis results, which show the amount of utilized resources and the maximum operating frequency of the decoder are presented in Table 9.

FPGA synthesis results of proposed digital image decoder have been compared with synthesis results of various state-of-the-art architectures of digital image decoders. The results of comparison are presented in Table 10.

It can be seen that the proposed hardware architecture for digital image decoder requires at least 32% less memory resources in comparison to the other state-of-the-art decoders which can process HD frame size or HD tile size. Some state-of-the-art architectures which process frame size or tile size smaller than HD size require total memory size lower than the memory size of the proposed solution. However, when frame size or tile size is taken into account as well, it can be concluded that proposed digital image decoder architecture can process 7.9 times larger frame/tile size, while it only requires 29% greater memory size in comparison with [2]. Similarly, the proposed digital image decoder can process 5.1 times larger frame size while it requires only a 3.1 times greater memory size in comparison [3]. The proposed solution for digital image decoder can process 5.1 times larger frame/tile size than [5] but utilizes only 15% more memory resources. Finally, the proposed digital image decoder architecture can process 32.4 times larger frame size, while requires only 64% greater memory size in comparison with 2 × 2 NoC decoder from [10]. In comparison to all other state-of-the-art solutions, the proposed architecture requires less memory size although it can process larger frame size.

Additionally, it can be seen that the proposed solution for digital image decoder has lower maximum operating frequency than architectures from [5,8], but can operate at higher frequency than all other state-of-the-art architectures.

## 6. Conclusions

The digital image decoder for efficient hardware implementation presented in this paper has many advantages in comparison to state-of-the-art solutions. The proposed entropy decoder and decoder probability estimator for efficient hardware implementation reduces the hardware complexity compared to the other state-of-the-art solutions by reducing or completely eliminating multiplication and division operations. The hardware complexity of the proposed dequantizer is reduced, in comparison to the state-of-the-art solutions, due to using the multiplication operation by power of two (which is implemented by using permanently shifted hardware connections between input and output bit lines), and due to using the multiplication operation with narrow-range integer which is implemented by simple lookup table. The proposed novel hardware realization of the inverse subband transformer, which performs 2-D inverse 5/3 DWT, utilizes 20% less memory resources compared to the best realization so far published in the literature. As a basic building block for the 2-D inverse 5/3 DWT, non-stationary hardware realization of the 1-D inverse 5/3 DWT filter has been used. This realization utilizes the lowest number of logic elements and the lowest number of registers, has the lowest total power dissipation and allows the highest operating frequency in comparison to any other realizations from the literature. The proposed digital image decoder requires at least 32% less memory resources in comparison to the other state-of-the-art decoders from the literature which can process HD frame size and requires effectively lower memory size than state-of-the-art solutions which process frame size or tile size smaller than HD size. The presented solution for digital image decoder has maximum operating frequency comparable with the highest maximum operating frequencies among the state-of-the-art solutions.

Future work on proposed digital image decoder would include modifications and optimizations which would increase maximum operating frequency while maintaining the reduced amount of utilized logical and memory resources. Additionally, future work could include the upgrade of proposed digital image decoder for efficient hardware implementation so that it can support decompression of ultra-high-definition (UHD) resolution images.

## Figures and Tables

**Table 1:** Decoding the limit of the samples of the components of the decomposed signal.

Limits of the Samples (Inclusive)		Range of the Samples	*MS*
Lower	Upper		
0	0	1	0
1	1	1	1
2	2	1	2
3	3	1	3
4	5	2	4
6	7	2	5
8	11	4	6
12	15	4	7
16	23	8	8
24	31	8	9
32	47	16	10
48	63	16	11
64	95	32	12
96	127	32	13
128	191	64	14
192	255	64	15
256	383	128	16
384	511	128	17
512	767	256	18
768	1023	256	19
1024	1535	512	20
1536	2047	512	21
2048	3071	1024	22
3072	4095	1024	23
4096	6143	2048	24
6144	8191	2048	25
8192	12,287	4096	26
12,288	16,383	4096	27
16,384	24,575	8192	28
24,576	32,767	8192	29
32,768	49,151	16,384	30
49,152	65,535	16,384	31

**Table 2:** CTX table for translating ternary context TC values into sign context SC.

*S*_0_	*S*_1_	*S*_2_	*S*_3_	*TC*	*SC*	*S*_0_	*S*_1_	*S*_2_	*S*_3_	*TC*	*SC*	*S*_0_	*S*_1_	*S*_2_	*S*_3_	*TC*	*SC*
0	0	0	0	0	0	1	0	0	0	27	2	2	0	0	0	54	1
0	0	0	1	1	0	1	0	0	1	28	1	2	0	0	1	55	0
0	0	0	2	2	0	1	0	0	2	29	2	2	0	0	2	56	0
0	0	1	0	3	4	1	0	1	0	30	0	2	0	1	0	57	1
0	0	1	1	4	1	1	0	1	1	31	1	2	0	1	1	58	0
0	0	1	2	5	2	1	0	1	2	32	1	2	0	1	2	59	2
0	0	2	0	6	1	1	0	2	0	33	1	2	0	2	0	60	0
0	0	2	1	7	0	1	0	2	1	34	0	2	0	2	1	61	0
0	0	2	2	8	1	1	0	2	2	35	0	2	0	2	2	62	0
0	1	0	0	9	0	1	0	0	0	36	1	2	0	0	0	63	0
0	1	0	1	10	1	1	1	0	1	37	1	2	1	0	1	64	0
0	1	0	2	11	1	1	1	0	2	38	1	2	1	0	2	65	0
0	1	1	0	12	1	1	1	1	0	39	0	2	1	1	0	66	0
0	1	1	1	13	0	1	1	1	1	40	1	2	1	1	1	67	1
0	1	1	2	14	0	1	1	1	2	41	0	2	1	1	2	68	1
0	1	2	0	15	0	1	1	2	0	42	1	2	1	2	0	69	0
0	1	2	1	16	4	1	1	2	1	43	0	2	1	2	1	70	0
0	1	2	2	17	0	1	1	2	2	44	0	2	1	2	2	71	3
0	2	0	0	18	4	1	2	0	0	45	3	2	2	0	0	72	0
0	2	0	1	19	2	1	2	0	1	46	0	2	2	0	1	73	4
0	2	0	2	20	2	1	2	0	2	47	1	2	2	0	2	74	3
0	2	1	0	21	1	1	2	1	0	48	1	2	2	1	0	75	2
0	2	1	1	22	0	1	2	1	1	49	2	2	2	1	1	76	2
0	2	1	2	23	3	1	2	1	2	50	2	2	2	1	2	77	0
0	2	2	0	24	0	1	2	2	0	51	0	2	2	2	0	78	2
0	2	2	1	25	0	1	2	2	1	52	0	2	2	2	1	79	1
0	2	2	2	26	1	1	2	2	2	53	1	2	2	2	2	80	0

**Table 3:** NEG table for inversion of the sign S.

*TC*	*P*(0)	*P*(1)	*NS*	*TC*	*P*(0)	*P*(1)	*NS*	*TC*	*P*(0)	*P*(1)	*NS*
0	0.5276	0.4724	0	27	0.6147	0.3853	0	54	0.4168	0.5832	1
1	0.5333	0.4667	0	28	0.4170	0.5830	1	55	0.5012	0.4988	0
2	0.4901	0.5099	1	29	0.6326	0.3674	0	56	0.5302	0.4698	0
3	0.2961	0.7039	1	30	0.4889	0.5111	1	57	0.5467	0.4533	0
4	0.4321	0.5679	1	31	0.4176	0.5824	1	58	0.5061	0.4939	0
5	0.6300	0.3700	0	32	0.4469	0.5531	1	59	0.4039	0.5961	1
6	0.4463	0.5537	1	33	0.5505	0.4495	0	60	0.5024	0.4976	0
7	0.4754	0.5246	1	34	0.5240	0.4760	0	61	0.4613	0.5387	1
8	0.4397	0.5603	1	35	0.4731	0.5269	1	62	0.4837	0.5163	1
9	0.5012	0.4988	0	36	0.4299	0.5701	1	63	0.5106	0.4894	0
10	0.5796	0.4204	0	37	0.5880	0.4120	0	64	0.5440	0.4560	0
11	0.4117	0.5883	1	38	0.5806	0.4194	0	65	0.5343	0.4657	0
12	0.5842	0.4158	0	39	0.4698	0.5302	1	66	0.4918	0.5082	1
13	0.5457	0.4543	0	40	0.4119	0.5881	1	67	0.4521	0.5479	1
14	0.5364	0.4636	0	41	0.5193	0.4807	0	68	0.5841	0.4159	0
15	0.5243	0.4757	0	42	0.4539	0.5461	1	69	0.5211	0.4789	0
16	0.7224	0.2776	0	43	0.4871	0.5129	1	70	0.4783	0.5217	1
17	0.5050	0.4950	0	44	0.4953	0.5047	1	71	0.6651	0.3349	0
18	0.7235	0.2765	0	45	0.3502	0.6498	1	72	0.4561	0.5439	1
19	0.3963	0.6037	1	46	0.4688	0.5312	1	73	0.6998	0.3002	0
20	0.6019	0.3981	0	47	0.5802	0.4198	0	74	0.6531	0.3469	0
21	0.4508	0.5492	1	48	0.4432	0.5568	1	75	0.6163	0.3837	0
22	0.5286	0.4714	0	49	0.3927	0.6073	1	76	0.5956	0.4044	0
23	0.6598	0.3402	0	50	0.6199	0.3801	0	77	0.5022	0.4978	0
24	0.4770	0.5230	1	51	0.5357	0.4643	0	78	0.6148	0.3852	0
25	0.5417	0.4583	0	52	0.4830	0.5170	1	79	0.4368	0.5632	1
26	0.4398	0.5602	1	53	0.4464	0.5536	1	80	0.5065	0.4935	0

**Table 4:** Implementation of division operation with numbers 3, 5, 7, 9, 11, 13 and 15.

Divide by [Decimal Number]	Multiply by [Hexadecimal Number]	Right Shift for [Binary Digits]
3	0AAAAAAAB	1
5	0CCCCCCCD	2
7	049249249	1
9	038E38E39	1
11	0BA2E8BA3	3
13	04EC4EC4F	2
15	088888889	3

**Table 5:** Number of multiplication and division operations per decoded symbol for $Total\neq 2^{w_{3}}$.

Operation Type	State-of-the-Art Range Decoder	$\mathrm{Proposed}\;\mathrm{Range}\;\mathrm{Decoder}\;r=V\cdot 2^{l}$		
		V=1	V=3 V=5	V≥7
Multiply	2	0	1	3
Divide	2	1	1	1

**Table 6:** Number of multiplication and division operations per decoded symbol for $Total=2^{w_{3}}$.

Operation Type	State-of-the-Art Range Decoder	$\mathrm{Proposed}\;\mathrm{Range}\;\mathrm{Decoder}\;r=V\cdot 2^{l}$		
		V=1	V=3 V=5	V≥7
Multiply	2	0	1	3
Divide	1	0	0	0

**Table 7:** FPGA synthesis results of various implementations of 5/3 filter on Altera FPGA EP4CE115F29C7.

1-D Inverse DWT 5/3 Filter @ 85 °C Unrestricted Frequency	Convolution [27,28,29,30,31,32]	Lifting [33,34,35,36,37,38]	Proposed
Total logic elements	234	120	120
Total registers	139	72	48
Critical path delay [ns]	5.4	8.2	5
Max frequency [MHz]	197.7	128	212
Total power dissipation [mW] @ 80MHz	132.4	134.4	130.9

**Table 8:** Comparison of various 2-D inverse 5/3 DWT architectures.

Architecture	On-Chip Memory Capacity	Off-Chip Memory Capacity
Non-separable [37]	10N	0
SIMD [37]	$N^{2}$	0
Direct [38]	$N^{2}$	0
Systolic-parallel [38]	14N	0
[28]	5N	$N^{2}/4$
[40]	$7N(1-2^{-J})$	0
[36]	$N^{2}+4N$	0
RA [39]	6N	0
FA [41]	3.5N	$N^{2}/4$
PA [41]	$6N(1-2^{-J})+0.5N$	0
[42]	≈7N	0
[43]	$8N(1-2^{-J})$	0
[44]	≈6.25N	0
[45]	2N	$N^{2}/2$
[46]	2N	$N^{2}/4$
[46]	$3N(1-2^{-J})+2N$	0
[47,48]	9N	0
Proposed	$4N(1-2^{-J})$	0

**Table 9:** FPGA synthesis results of presented digital image decoder.

Parameter	Value
Number of logic elements	77,127
Memory size	1,884,207 bits
Number of multipliers	12
Maximum operating frequency at 85 °C	114.71 MHz

**Table 10:** Comparison of FPGA synthesis results for various architectures of digital image decoder.

Architecture	Frame Size/ Tile Size	Memory Size [kbits]	Maximum Operating Frequency [MHz]
[1]	160 × 120	n/a	24.15
[2]	512 × 512	1424	89.9
[3]	704 × 576	594	105.6
[4]	1920 × 1080	433,357	42.8
[5]	512 × 512	1602	116.9
[6]	2048 × 1080	2710	n/a
[7]	1920 × 1080	6192	n/a
[8]	1920 × 1080	5182	180
[9]	1920 × 1080	3277	110
[10] 3 × 3 NoC	320 × 200	1842	n/a
[10] 2 × 2 NoC	320 × 200	1125	n/a
Proposed	1920 × 1080	1840	114.71

## Data Availability

Data available on request.
